# Characterizing diversity of food systems in view of sustainability transitions. A review

**DOI:** 10.1007/s13593-018-0550-2

**Published:** 2018-12-17

**Authors:** Daniel Gaitán-Cremaschi, Laurens Klerkx, Jessica Duncan, Jacques H. Trienekens, Carlos Huenchuleo, Santiago Dogliotti, María E. Contesse, Walter A. H. Rossing

**Affiliations:** 1 Farming Systems Ecology Group, Wageningen University and Research, PO Box 430, 6700AK Wageningen, The Netherlands; 2 Knowledge, Technology and Innovation Group, Wageningen University, PO Box 8130, 6700EW Wageningen, The Netherlands; 3 Rural Sociology Group, Wageningen University and Research, PO Box 8130, 6700EW, Wageningen, The Netherlands; 4 Business Management and Organisation Group, Wageningen University and Research, PO Box 8130, 6700EW Wageningen, The Netherlands; 5 Escuela de Agronomía, Pontificia Universidad Católica de Valparaíso, Calle San Francisco SN, La Palma, Quillota, 2260000 Chile; 6 Facultad de Agronomía, Universidad de la República, Av. Garzón 780, 11200 Montevideo, Uruguay

**Keywords:** Food system, Sustainability transitions, System diagnosis, Agricultural innovation systems, Agricultural production systems, Value chains, Grassroots movements, Agroecology, Food regime, Transformations

## Abstract

Dominant food systems are configured from the productivist paradigm, which focuses on producing large amounts of inexpensive and standardized foods. Although these food systems continue being supported worldwide, they are no longer considered fit-for-purpose as they have been proven unsustainable in environmental and social terms. A large body of scientific literature argues that a transition from the dominant food systems to alternative ones built around the wider principles of sustainable production and rural development is needed. Promoting such a sustainability transition would benefit from a diagnosis of food system types to identify those systems that may harbor promising characteristics for a transition to sustainable food systems. While research on food system transitions abounds, an operational approach to characterize the diversity of food systems taking a system perspective is still lacking. In this paper we review the literature on how transitions to sustainable food systems may play out and present a framework based on the Multi-Level Perspective on Socio-Technical Transitions, which builds upon conceptual developments from social and natural science disciplines. The objectives of the framework are to (i) characterize the diversity of existing food systems at a certain geographical scale based on a set of structural characteristics and (ii) classify the food systems in terms of their support by mainstream practices, i.e., dominant food systems connected to regimes; deviate radically from them, niche food systems such as those based on grassroots innovation; or share elements of dominant and niche food systems, i.e., hybrid food systems. An example is given of application of our framework to vegetable food systems with a focus on production, distribution, and consumption of low-or-no pesticide vegetables in Chile. Drawing on this illustrative example we reflect on usefulness, shortcomings, and further development and use of the diagnostic framework.

**Contents**

- 1. Introduction
- 2. Characterizing food system diversity: a framework
- 2.1. The Multi-Level Perspective (MLP) framework
- 2.2. Food systems through the lens of the Multi-Level Perspective (MLP)
- 2.3. Characterizing the diversity of food systems
- 3. Methodological approach for implementation of the framework
- 3.1. Step 1: identifying the food system and defining the system boundaries
- 3.2. Step 2: identifying agricultural production system types
- 3.3. Step 3: identifying the types of value chains associated to agricultural production system types
- 3.4. Step 4: identifying the multiple setups of support structures
- 3.5. Step 5: identifying the diversity of food systems
- 3.6. Step 6: assessing food system outcomes
- 3.7. Step 7: classifying food systems through the lens of the MLP
- 4. Illustration to vegetable food systems in Chile: lessons from the application of the framework
- 4.1. Data collection and analysis
- 4.2. Characterization of vegetable food systems in Chile and food system outcomes
- 4.3. Classification of vegetable food systems in Chile
- 5. Conclusions
- Acknowledgments
- References

## Introduction

Food systems are generally conceived as the network of actors and activities that interact with one another, within an ecological, social, political/cultural, and economic environment. Activities include growing, processing, distributing, consuming, and disposing of foods, from provision of inputs to waste and recycling (Ericksen 2008; IPES-Food 2015). Beyond the actors involved directly in these activities, food systems also comprise the structural conditions (e.g., rules, standards, policies), and dedicated agents (e.g., actors in public and private organizations such as extension services and research) that support daily operation as well as continuous optimization and innovation of the systems (IPES-Food 2015). Multiple interactions between the actors, activities, structural conditions, and dedicated agents lead to different configurations of food systems, which can be linked to multiple co-existing production/consumption paradigms and values (e.g., productivist paradigm, life science paradigm, ecological and health paradigm, re-localization paradigm) (Lamine 2015; Lang and Heasman 2015; Plumecocq et al. 2018). The configuration of a food system influences its performance in terms of three normative food system goals, i.e., food security and nutrition, environmental security, and social welfare (Ingram 2011).

Historically, the global food system has been ordered by specific rules and structures governing the production, circulation, and consumption of food on a world scale, which organize the accumulation of capital in the agri-food sector (McMichael 2009). This is what Friedmann and McMichael (1989) have conceptualized as “food regimes,” periods and patterns in capitalist history where agriculture has played a strategic role. At present, many food systems in different countries are in one way or another influenced by the established food regime, which McMichael (2009) calls the corporate food regime, for example, influenced by current international patterns of trade and the increasing emergence of agro-business corporations and industries that often determine what farmers produce and how value added is distributed (Clapp 2018; O'Kane 2012; Therond et al. 2017). The corporate food regime embodies the productivist paradigm rooted in the green revolution, in which food systems enact an industrial approach to food and farming, with state and industry support primarily geared to producing large amounts of standardized foods (Lang and Heasman 2015; Lowe et al. 1993; Therond et al. 2017), often leaving aside environmental and societal food system goals (Dobermann et al. 2013; IPES-Food 2015; O'Kane 2012). The productivist paradigm to which many food systems adhere has resulted in strong negative environmental and social impacts around the world (Baroni et al. 2006; Black et al. 2011; Ericksen 2008; Tittonell et al. 2016). On top of these impacts, some have argued that the dominant food systems, which are the food systems aligned to the corporate food regime, are ineffective at feeding the world population (Tittonell et al. 2016), of which still close to 800 million people go hungry and over 1 billion are overweight (FAO, UNICEF, WFP, WHO 2017).

A wide body of scientific literature has argued that so-called sustainability transitions are needed to enable a transformation from the existing corporate food regime to an alternative regime configured around the wider principles of sustainable production and rural development (Brunori et al. 2013; Hinrichs 2014; Holt-Giménez and Altieri 2013; Holt-Giménez and Shattuck 2011; Hubeau et al. 2017; Ingram 2015; Meynard et al. 2017). Within this body of work studies look at different issues in agricultural and food system sustainability transitions such as transformative change agency, science-driven and grassroot transition movements, stability of the food regime and lock-in, and the interaction between innovation networks and the incumbent food regime (see, for example, Bui et al. 2016; Diaz et al. 2013; Elzen et al. 2004; Ingram 2015; Ingram and Maye 2016; Klerkx et al. 2010; Lamine 2011; Lamine et al. 2012; Levidow et al. 2014; Meynard et al. 2017; Rossi 2017; van der Ploeg et al. 2004; Vanloqueren and Baret 2008; Vlahos et al. 2017; Wilson and Tisdell 2001). Such sustainability transitions may follow different pathways in enacting different alternative paradigms for shaping future food systems (Pigford et al. 2018; Plumecocq et al. 2018). Whichever sustainability transition pathway is chosen (Roep and Wiskerke 2004; Plumecocq et al. 2018), a common aspect of any pathway is that it requires coupled innovations in technologies (e.g., agronomic practices, processing and recycling technologies) and in non-technological domains (e.g., cooperation between food system actors, different upstream and downstream organizational arrangements, and consumption practices), in activities of growing, processing, distributing, consuming, and disposing of foods (Meynard et al. 2017), as well as dedicated change agents and networks that promote these transitions (e.g., grassroot social movements such as in agroecology) (Bui et al. 2016; Klerkx et al. 2010, 2012; Lamine et al. 2012; Roep et al. 2003) to deal with power structures in large systems (Dentoni et al. 2017; Pigford et al. 2018).

**Fig. 1:**
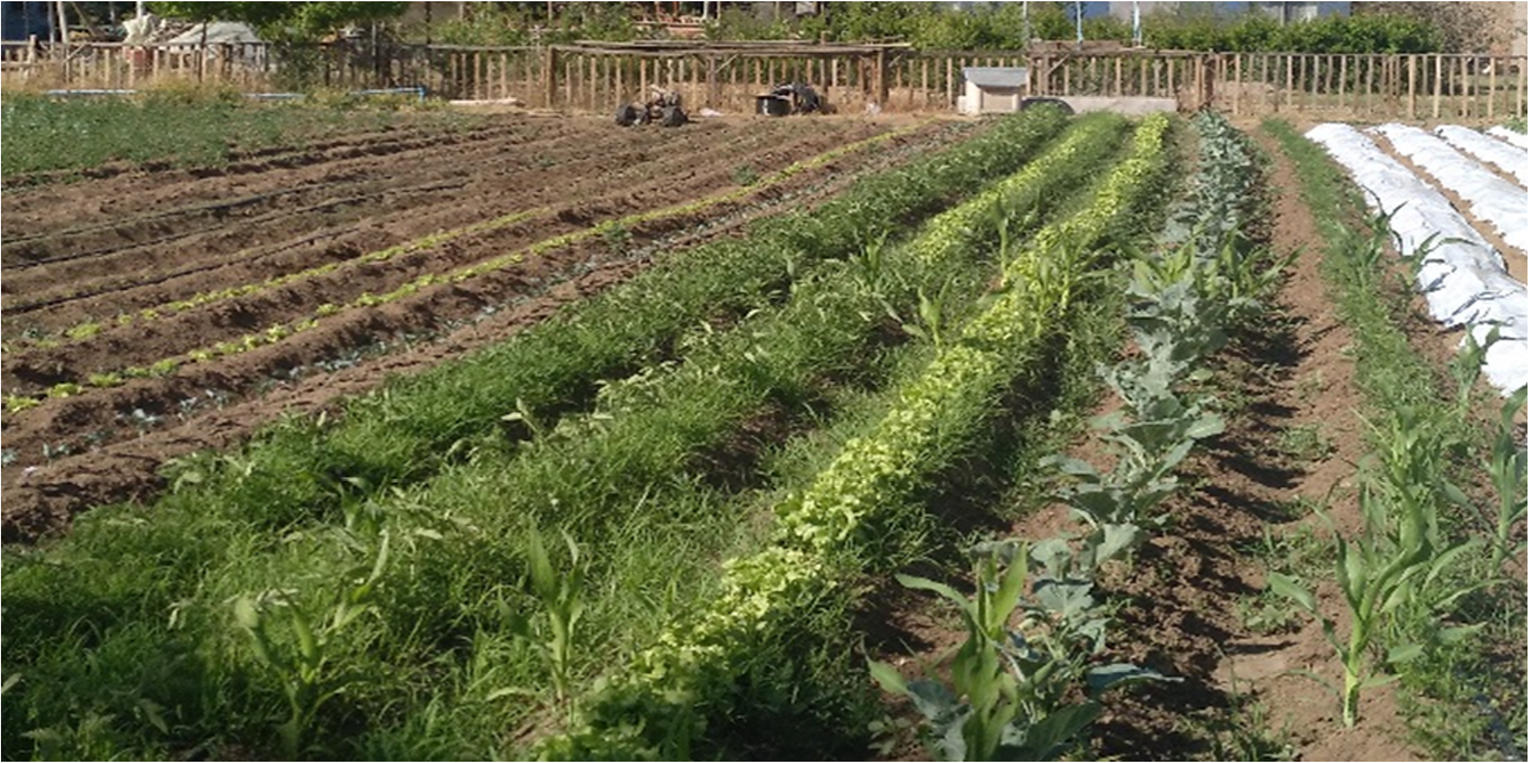
Vegetable field in Chile representing a move away from the productivist paradigm towards agroecological production systems

A number of alternative paradigms proposing sets of technological and non-technological innovations to foster sustainability transition pathways have emerged that can inspire and enable the redesign of current dominant food systems. A first category comprises a set of innovations representing a move towards the ecological modernization of food systems by reconciling agriculture, food production, and the environment. Examples include sustainable intensification (Garnett et al. 2013), input-substitution production systems (Singh et al. 2011), climate smart agriculture (Lipper et al. 2014), precision agriculture (Gebbers and Adamchuk 2010; Rains et al. 2011), eco-efficiency (Carberry et al. 2013), environmental-friendly food-processing technologies (Barbosa- Cánovas and Gould 2000), and food-packaging alternatives (Han 2013). Although commonly advocated as “sustainable,” some commentators see many of these innovations as incremental, only perpetuating current industrialized modes of production, distribution, and consumption of foods (Loos et al. 2014; Maye and Duncan 2017; Rosset and Altieri 1997). These incremental innovations have been argued to be associated with the selective appropriation by dominant food systems of the ecological and health agendas of social and environmental movements. Friedmann (2005) elaborates on this idea by suggesting the possible emergence of a food regime named as the “corporate-environmental food regime.” A second set of innovations may exemplify a more radical move away from the productivist paradigm towards multi-functional and ecological agricultural production systems (Doré et al. 2011; Duru et al. 2015; Tittonell et al. 2016), and decentralized, differentiated, localized, and ecological value chains (Feagan 2007; Maye and Kirwan 2010; Tregear 2011), which are supported by quality conventions embedded in trust, tradition, and place (Feagan 2007) (Fig. 1). These innovations seek to overcome “business-as-usual” solutions to sustainability issues by reshaping food practices, not only from a technical perspective but also through changes in social interactions and modes of organization (Lamine et al. 2012). These innovations include, among others, production of food based on ecological intensification (Doré et al. 2011; Tittonell et al. 2016) and biodiversity-based agriculture (Duru et al. 2015), supported by multiple forms of alternative food networks (AFNs) such as community-supported agriculture (CSA), food cooperatives, farmers’ markets, or box schemes (Renting et al. 2003). These innovations in food systems could play a radical role in the transition from the corporate food regime towards a new and more sustainable regime (Holt-Giménez and Shattuck 2011).

While there is thus a growing body of research in the sustainability transition literature interested in food systems, we identify two knowledge gaps. First, although this body of research makes reference to sustainability transition in food systems, a “whole food systems” approach still remains limited (Markard et al. 2012). The focus is commonly on the farm component of the food system without putting due attention to coupled innovations in all other components that are part of the food system. Second, research have often focused on unraveling transition dynamics within certain projects or innovation niches that work on alternative food systems. There is still a lack of an operational approach that enables a diagnosis in a given country or region about what are the (a) various dominant food systems and (b) the alternatives to dominant ones that may harbor promising innovations for improving or changing unsustainable systems and that are creating new contexts of opportunities for a transition process. Such an approach could be both useful to inform research on food regimes and food system transitions, as well as policy makers to guide investments and see how they can orient their innovation policies to support certain desired transition pathways and counteract undesired dominant systems (following Kivimaa and Kern 2016).

In this paper both literature gaps are addressed by presenting an integrated and structured conceptual and methodological framework that provides a diagnostic tool that enables a characterization of the diversity of food systems that co-exist within a given geographical area such as a country, in order to identify patterns of more and less sustainable characteristics. This framework takes into account the multi-dimensional characteristics of food systems and, therefore, integrates and builds upon existing concepts in agronomy, value chain management, innovation systems, food system governance, and environmental sciences. It complements other frameworks which for example look at food system sustainability performance metrics (Zurek et al. 2018). The operational objectives of our framework are to (i) enable a characterization of food systems based on their structural characteristics and (ii) enable a classification of the food systems from dominant food systems to niche food systems and hybrid forms. After describing our framework (Sect. 2), this paper introduces a methodological approach that combines multiple methods of data collection for implementation of the framework (Sect. 3). To illustrate how the framework may be operationalized, an example is given of application of our framework to vegetable food systems in Chile with a focus on production, distribution, and consumption of low-or-no pesticide vegetables. Drawing on this illustrative example we reflect on the usefulness, shortcomings, and further development and use of the framework (Sects. 4 and 5).

## Characterizing food system diversity: a framework

### The multi-level perspective framework

**Fig. 2:**
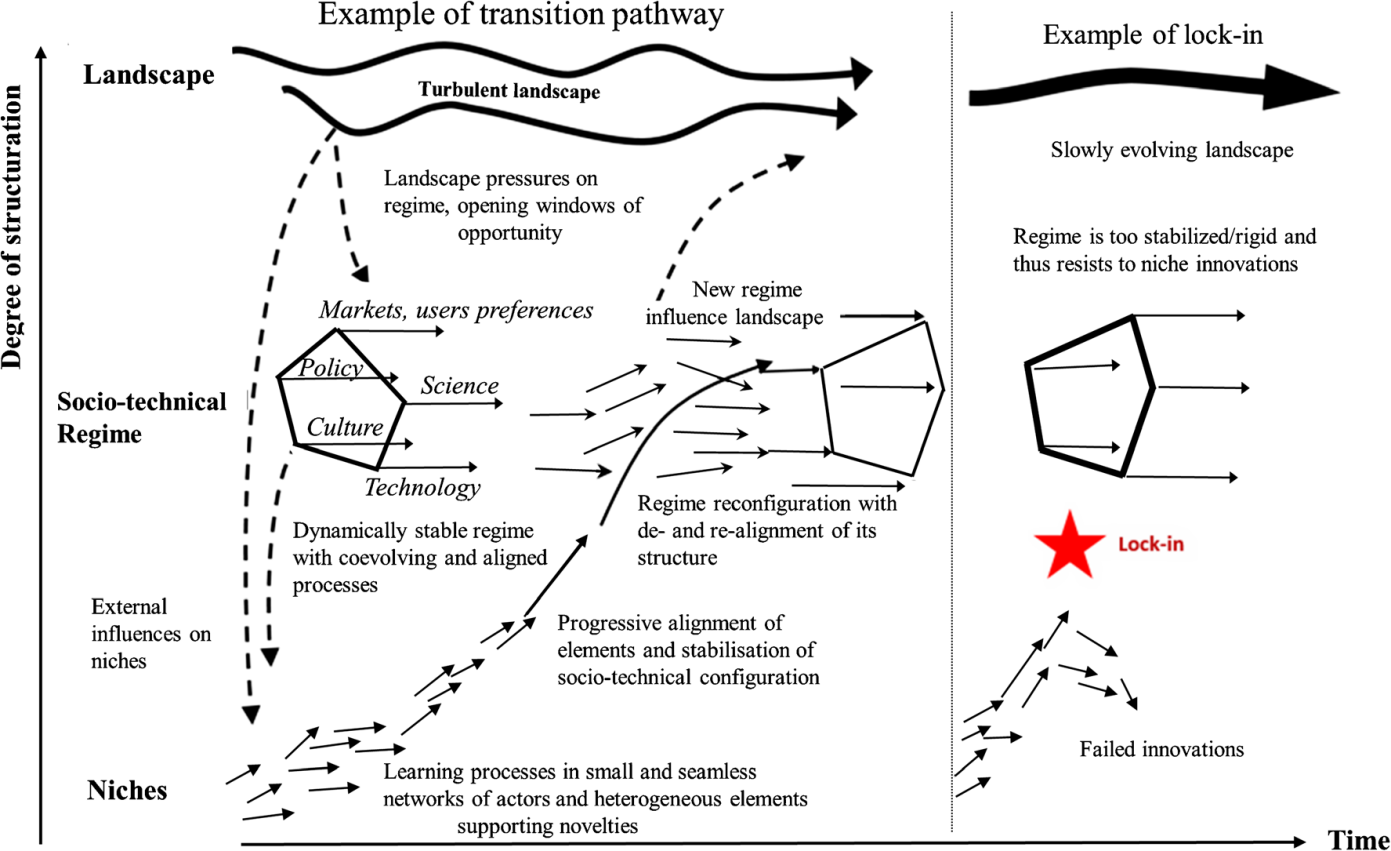
The multi-level view of transitions (Geels 2002). Adapted from Ollivier et al. (2018)

The food regime perspective has been widely used to understand the global food system and its crisis as part of a broader historical understanding of the global drivers and the geo-political and economic conditions (McMichael 2009), using essentially a global political economy approach (Pereira and Drimie 2016). It seems to be less appropriate to unravel the dynamics of the multiple coupled innovation processes in food systems at lower spatial levels, i.e., regional or national. These are specifically captured by another prominent approach, which has emerged in parallel to analyze sustainability transitions in food systems, i.e., how sustainability innovations emerge, scale out, and influence the way food systems are configured. This approach is the so-called multi-level perspective (MLP) proposed by Geels (2002), which has been applied to sectors such as energy, transport, and food (Hinrichs 2014). Generally speaking, the MLP proposes that transitions emerge from the complex interplay of processes occurring at, and between, three intertwined levels: (1) the socio-technical regime (meso-level), (2) the niches (micro-level), and (3) the socio-technical landscape (macro-level) (Fig. 2).

The MLP definition of socio-technical regime comes from a tradition of looking at the evolutionary character of coupled socio-technological change and refers to socio-technical regimes as coherent sets of social and technological elements that underpin basic societal functions (Holtz et al. 2008), among them, the production, commercialization, and consumption of food. This notion of regime postulates that a given system is locked-in by path dependency and stability (Wieczorek 2018), and therefore, it seeks to maintain its dominant position generally by favoring trajectories of incremental adjustments to fix problems within the regime (Ingram 2015). Due to its stabilizing features, the socio-technical regime generally blocks the emergence of radical innovations that challenge the rules about how the system operates (Ingram 2015; Meynard et al. 2017). Such radical innovations are commonly generated in niches (Geels 2002). The niches are alternative socio-technical systems that provide a protected space for development of new technologies, new concepts, and new ways of organization and of doing things (van der Ploeg et al. 2004). In the MLP, it is acknowledged that tensions within the socio-technical regime as well as exogenous macro-trends (e.g., climate change, occurrence of earthquakes, droughts, or hurricanes) and endogenous macro-trends (socio-technical regimes pertaining to other sectors, e.g., energy, health, tourism, and mobility) create pressure on both niches and socio-technical regimes, and provide a space for change (Avelino 2017). These macro-trends are referred to as the socio-technical landscape. Interactions between the niche, regime, and landscape levels lead to a whole set of transition pathways. These pathways emanate from efforts by the niche actors in collaboration with regime actors or from the regime itself (Ingram 2015; Klerkx et al. 2010) and can be of a more incremental or radical nature (Ingram 2015).

### Food systems through the lens of the MLP

Characterizing the diversity of co-existing food systems in a certain geographical area fits well with the MLP. The socio-technical regime manifests in the way dominant food systems are configured and how they perform (Ingram 2015). The technical elements of the regime in case of food systems include physical inputs, plant-breeding techniques, harvesting technologies, transport and logistics, food processing, and recycling technologies. The social elements involve the prevailing attitudes towards farming, the conception of sustainability, ideas about nutritional value, policy measures, price-support mechanisms, and the organized interests, among many others (Ingram 2015; Smith et al. 2010). To distinguish it from the concept of “food regimes” sensu Friedmann and McMichael (1989), and to make it more applicable to food systems analysis at, for example, country level, we use the term “food system regime.” The rules and structures of the corporate food regime manifest in a given country dependent on the particular actors and the biophysical, infrastructural, and institutional conditions in that country. A food system regime in a country typically represents mainstream social and technical elements dominated by conventional industrial farming and value chains controlled by large-scale and powerful agri-food industries and companies (Morrissey et al. 2014; Pitt and Jones 2016). This food system regime may also include structures and rules for the greening of food production, retailing, and consumption, as was the case for organic agriculture that has become conventionalized and industrialized in last decades (Darnhofer et al. 2010).

Tensions within the food system regime and ongoing landscape pressures (the macro-level in the MLP) destabilize the food system regime and create opportunities for innovations to emerge both within the dominant food systems, which are aligned and supported by the food system regime, and in niches (the micro-level in the MLP) (Avelino 2017; Smith et al. 2010). For example, the impacts of climate change on agriculture may open the space for the adoption of (novel) farming practices that allow adapting agriculture to extreme weather events. As a second example, the pressures of social movements and consumers advocating more healthy food may create the opportunity for closer farmer-consumer relationships or may lead to adoption of new food safety control systems throughout the value chain. The food system regime is often locked-in on generating incremental innovations that solve problems within the regime. The food system regime functions to maintain the status quo and therefore to marginalize or co-opt more radical innovations (Ingram 2015). These radical innovations are often developed in niches, spaces in which the collective action of diverse actors is facilitated to develop multiple alternative solutions to advance to more sustainable ways of producing, commercializing, and consuming of food (Klerkx et al. 2010 in Ingram 2015; Pigford et al. 2018).

A transition in food systems can occur when niches interact with the food system regime. Innovations in niches can fit-and-conform to the food system regime or they can stretch-and-transform it (see Smith and Raven (2012) for a detailed description of fit-and-conform and stretch-and transform). Whether the niches fit-and-conform to the food system regime or stretch-and-transform it depends on different factors such as the niches’ internal dynamics, their interactions with the food system regime and with other niches, and their ambition to transform the food system regime (Darnhofer et al. 2015). A transition to sustainable food systems may not be entirely driven by niche actors but may also rely upon actors in the food system regime that champion different transition pathways and are capable of fostering these changes (Ingram 2015; Smith and Raven 2012). Considering that the actors and activities that constitute a food system are all strongly interconnected, both regime-induced and niche-induced innovations, either of an incremental or radical nature, may lead to multiple co-existing food systems within a country, i.e., configurations of dominant food systems that may share some elements but differ in others and are supported by the food system regime, and configurations of niche food systems that challenge the way the dominant food systems operate. As the boundaries between the dominant food systems and the niche food systems are blurry and permeable, hybrid food systems constituted by a mix of regime-induced and niche-induced innovations may also exist. These systems are organized and perform at the crossroads of the food system regime and the niche food systems (Lamine et al. 2012; Plumecocq et al. 2018), and often involve actors in the food system regime that are sympathetic to the innovations of the niche food systems (Darnhofer et al. 2015).

In the next section we introduce and describe the structural characteristics by which co-existing food systems can be characterized and can be classified as dominant food systems, niches, and hybrid forms.

### Characterizing the diversity of food systems

**Fig. 3:**
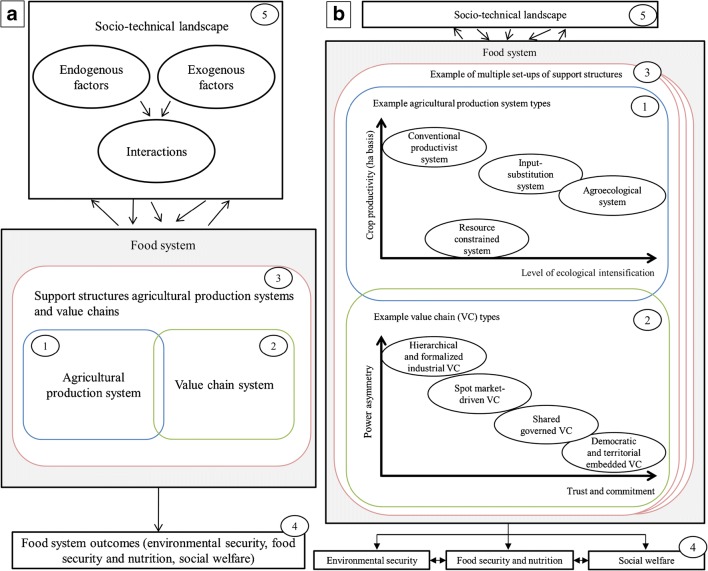
The food system. **a** Components of a food system: agricultural production system (number 1), value chain system (number 2), and structures for support of innovation and everyday functioning of agricultural production systems and value chains (number 3) and food system outcomes (number 4). The three components influence and are influenced individually or jointly by the socio-technical landscape (number 5). The conceptualized food system builds on Ericksen’s (2008) food system framework. **b** Heterogeneity within the three components of the food system. Illustration of the possible diversity of agricultural production systems (number 1) and diversity of value chains (VC) (number 2), which are embedded in multiple setups of structures for support of innovation and everyday functioning of agricultural production systems and value chains (number 3)

The framework presented here characterizes and maps the diversity of existing food systems in terms of their transition pathways, i.e., dominant food systems supported and aligned to the food system regime, niche food systems, and hybrid forms, based on a set of structural characteristics. These structural conditions have different configurations depending on whether they connect to the food system regime, niche food systems, and hybrid forms. A common point in different attempts to characterize food systems is that three interrelated food system components are distinguished: (i) the agricultural production system, (ii) the value chain, and (iii) the structures for support of innovation and everyday functioning of agricultural production systems and value chains (hereafter support structures) (Fig. 3a). The three components of a food system are individually and jointly influenced and (de)stabilized by the socio-technical landscape. In the long term the components of a food system may influence the socio-technical landscape. Final outcomes of the food system vary in terms of food security and nutrition, environmental security, and social welfare, depending on how the food system is configured (Fig. 3a).

The first component of a food system, the *agricultural production system*, comprises the farm structure and the set of agricultural practices that producers mobilize to transform land, capital, and labor into useful products that can be consumed or sold (Fresco and Westphal 1988; Boiffin et al. 2004 in Le Gal et al. 2011). The agricultural production system may comprise cropping and livestock systems that interact with the environment. The second component, the *value chain*, comprises the network of horizontally and vertically related value chain actors such as traders, wholesalers, processors, retailers, and exporters that operate jointly to bring agricultural products to consumers (Trienekens 2011), who, themselves, are also part of the value chain. Value is added by each activity of the chain (Schneemann and Vredeveld 2015). The horizontal dimension reflects the relationships between actors in the same value chain activity (e.g., marketing cooperatives, farmers’ associations, and collaborative agreements between processors). Vertical relationships reflect how value chain actors organize and coordinate themselves to bring the products from the primary producer to the final consumer (Trienekens 2011). To allocate and mobilize resources and to coordinate and control the horizontal and vertical relationships some form of governance is necessary (Provan and Kenis 2008). The third component, *the support structures*, refers to the structures that influence the creation, adoption, and dissemination of innovations (e.g., through fiscal incentives to R&D); provide support to agricultural producers and value chain actors to obtain information, skills, capabilities, and technologies to solve everyday problems; and enable various forms of interaction and learning processes at different geographic levels (Davis 2008; Edler and Fagerberg 2017). These structures are comprised by public and private research and development (R&D) activities and programs, extension services that include grassroot knowledge-sharing systems, and economic and innovation policy.

Agricultural production systems, value chains, and the support structures are diverse. An example of this heterogeneity is illustrated in Fig. 3b. Multiple setups of support structures co-exist, e.g., varying innovation policy mixes, multiple public and private R&D agendas, priorities to solve sustainability related issues, and different approaches for extension. Each of these setups of structures may either support innovation and everyday functioning of dominant modes of food production and value chains, and thus reproduce the current state of affairs, or they may provide the structural conditions to support the development of innovations of a more radical nature (Schut et al. 2015). Some structures of a given setup may also be shared or may be overlapped across different food systems. At the same time, a given setup may be a factor that constrains the development of innovations or causes the innovations to fail. For example, by subsidizing fossil fuel-based agrochemicals and commodity crops, governmental policies may promote food production in industrialized monoculture agricultural production systems that primarily benefit larger multi-national agribusiness (Kremen et al. 2012). This was the case, for example, for the EU Common Agricultural Policy that provided agricultural subsidies proportional to farmed area, thus favoring large-scale and industrial farming (IPES-Food 2016). At the same time, implementation of these policies creates obstacles to the adoption of more radical innovations in food production by disfavoring food that do not use these external inputs, e.g., those of agroecological and organic origin. Alternatively, support structures such as farmer field schools, agroecological policies, and NGOs as well as grassroot networks and social movements could bring about conditions for joint learning processes among food system actors for adoption and distribution of food products coming from ecologically intensive agricultural production systems. Hence, these multiple setups of support structures heavily influence how the agricultural producers and value chain actors build their strategies by adopting and/or developing technologies and modes of economic organization that best adapt to the environment.

Agricultural producers can opt or may be forced, as result of the growing concentration and power of downstream and upstream corporations and industries, to organize their systems following dominant modes of food production, which are based on strongly simplified crop sequences, standardized crop management, and systematic use of chemical inputs (Therond et al. 2017). At the other side of the spectrum, producers can opt to avoid or reduce their dependence on purchased inputs and follow ecologically more intensive approaches to food production such as agroecology, diversified production systems, some forms of organic agriculture, and permaculture (Tittonell 2014a). Between these two extremes, a continuum of agricultural production systems may co-exist. Some examples include agricultural production systems belonging to smallholders that are constrained by resources such as land, water, energy, phosphorous, and nitrogen, and input-substitution systems in which producers seek to replace some of the conventional chemical inputs with more “environmentally friendly inputs” while maintaining the principles and values of conventional agriculture (Therond et al. 2017). As an example, in Fig. 3b (number 1), agricultural production system types are distinguished by the level of ecological intensification of production and the attainable productivity on a per hectare basis (adapted from Tittonell et al. 2016). Many other variables may be mobilized to characterize agricultural production systems, e.g., area, provision of ecosystem services, labor, and mechanization.

In value chains, organization, coordination, and operation of downstream and upstream activities can take many forms. For example, value chain actors may take part in hierarchical value chains with administrative control; actors may join value chains with loose and non-exclusive relationships; actors may participate individually in value chains with little or no formal cooperation; or actors can opt to be organized collectively and operate in cooperation to address value chain requirements. As it is the case with producers, the ongoing concentration of the agri-food industry in many parts of the world can serve to limit the choices of actors to participate in certain value chains (see Weis 2007; Ros-Tonen et al. 2015; Kilelu et al. 2017 for a detailed description). As an example of the heterogeneity in the organization and operation of value chains, in Fig. 3b (number 2), the features power asymmetry between value chain actors and trust and commitment towards the chain are used to distinguish four types of value chains (adapted from Duncan and Pascucci 2017). In the example, value chain types range from hierarchical and formalized value chains, in which actors are often fully dependent on a specific value chain party (e.g., large processor or large retailer), to democratic and territorially embedded value chains that organize themselves at the community-level around trust and horizontal decision making. Many other variables may be mobilized to characterize value chains, e.g., transaction costs, size of the value chain, goal of each value chain actor, and asset specificity in value chains.

**Fig. 4:**
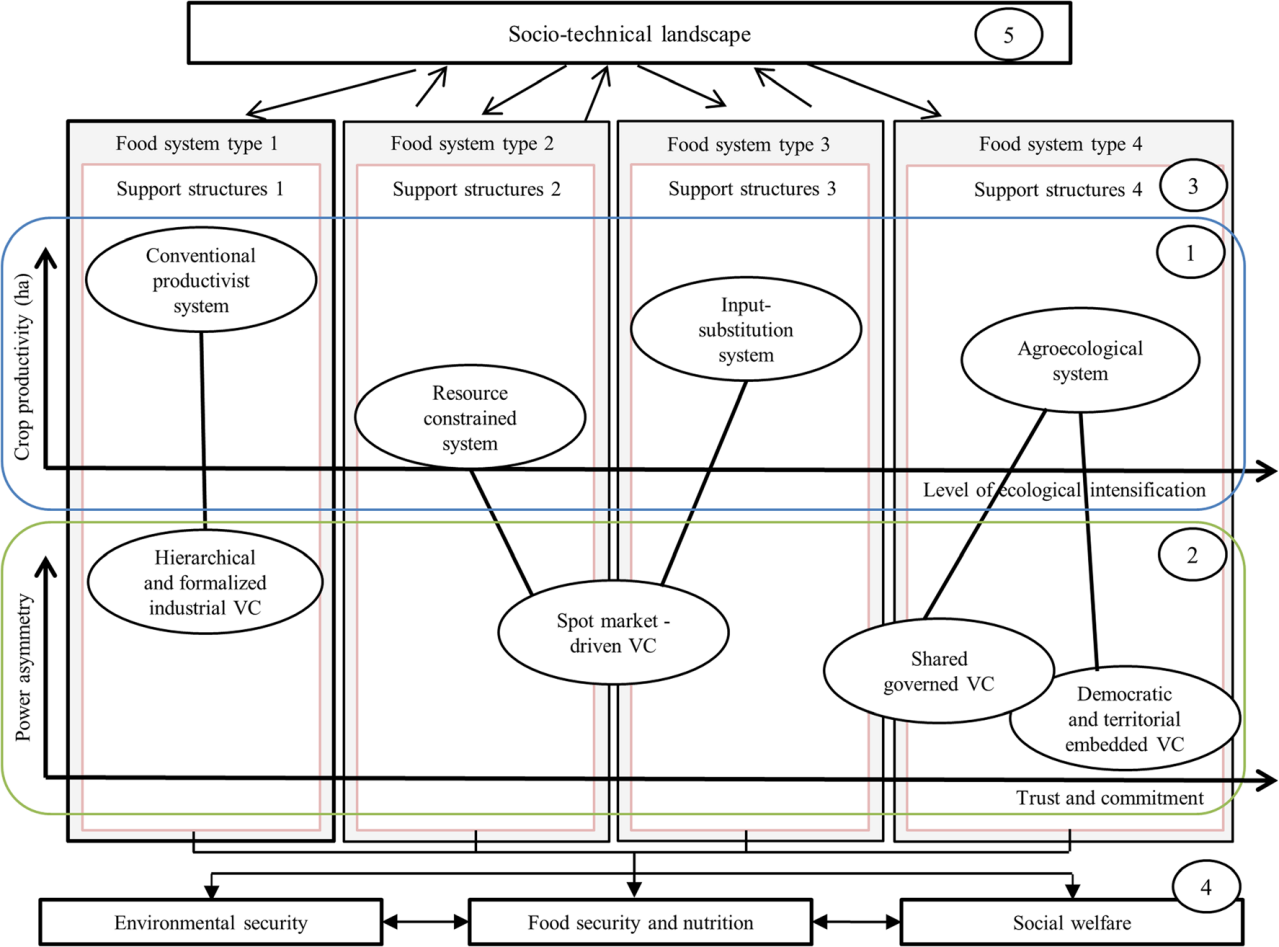
Example of co-existing food systems. Food systems result from the interrelation between (i) a type of the multiple agricultural production systems exemplified by the level of ecological intensification and the attainable productivity on a per hectare basis (adapted from Tittonell et al. 2016) (number 1), (ii) a type of the multiple value chain(s) that are exemplified based on the level of trust and commitment towards the chain and the power asymmetry between value chain actors (adapted from Duncan and Pascucci 2017) (number 2), and (iii) a setup of structures for support of innovation and everyday functioning of the agricultural production system and the associated value chain (number 3). Final outcomes of the food systems include food security and nutrition, environmental security, and social welfare (number 4). The food systems and their components are individually and jointly influenced by the socio-technical landscape (number 5)

Combining a type of agricultural production system with a type of value chain(s), along with their enabling and encompassing setup of support structures, results in multiple types of food systems that can be classified as dominant food systems, niche food systems, or hybrid forms. Dominant food systems will be those that are supported by mainstream practices in agricultural production systems and value chains, niche food systems will be those systems whose practices deviate radically from those that are found in the dominant food systems, and hybrid food systems will be those systems that represent heterogeneity and dissent within the food system regime. These systems are sympathetic to some niche innovations but are mainly constituted by mainstream practices. Hybrid systems may be promising in transition processes as they may foster broader processes of change, by creating linkages between the niche food systems and the food system regime. Building from Fig. 3b, an example of multiple food systems is illustrated in Fig. 4. In this example, a conventional productivist agricultural production system is connected to a hierarchical and formalized vertical value chain in which agricultural producers are either part of large agribusiness companies (e.g., input-supply companies, retailers, processors) or conform to their standards (food system type 1 in Fig. 4 may be classified as a dominant food system). At the other side of the spectrum, agroecological production systems may be either supported by shared-governed or democratic and territorially embedded value chains, which coordinate production and distribution activities on the basis of community relations and trust between producers and consumers (Sonnino and Marsden 2006). This can be the case of food systems that develop in niches supported by grassroot movements and social groups (food system type 4 in Fig. 4 can be classified as a niche food system).

Depending on how a food system is configured, performance in terms of satisfying food security and nutrition, environmental security, and social welfare varies. A food system contributes to food security and nutrition when it is able to provide consumers with sufficient, safe, and nutritious food. The contribution of a food system to environmental security involves the maintenance or enhancement of physical stocks of natural capital (e.g., land, soil, water, and biological resources) and the provision of ecosystem services (Kumar 2012). Social and economic outcomes of a food system, denoted as social welfare, encompass how the food system and its activities support livelihoods more broadly. Hence, social welfare performance may include, among others, sufficient income for every food system actor (farmers and processors, retailers), which requires a fair distribution of the benefits (FAO 2014a); autonomy and empowerment of food system actors and the communities in which the system is embedded; employment and fair labor conditions; and maintained and enhanced social capital (Ericksen 2008; FAO 2014a). Environmental security outcomes and social welfare outcomes co-determine food security and nutrition (Ericksen 2008). In the example of Fig. 4, food system type 1 may perform well in terms of economic outcomes and poorly in environmental and social welfare, whereas food system type 4 may emphasize environmental security and social welfare at the expense of economic outcomes.

## Methodological approach for implementation of the framework

We propose a seven-step procedure to characterize food systems and classify them as dominant food systems, niche food systems, or hybrid forms (Table 1). Each of the seven steps requires hybridizing methods of analysis and techniques of data collection. In the ideal case, implementation of the steps should combine diverse methods and techniques to generate both qualitative and quantitative data. Qualitative data provides the basis for the identification and description of agricultural production systems, value chains, and the support structures. Quantitative data provides the descriptive statistics and trends to complement the information gathered through the qualitative approaches (Schut et al. 2015). Combining different types of methods and data collection techniques works to enhance the credibility and strength of the analysis (Schut et al. 2015) and ensure corroboration, triangulation, and validation of data (Sandelowski 2000). The actual selection of the methods and techniques to be used depends on the available (economic and human) resources and time. In case of low availability of resources, a solely qualitative approach is sufficient if it is able to target different stakeholders individually and/or in groups across different levels with broad knowledge and expertise on the food systems under study. The objective of each of the seven steps and the associated methods and techniques are summarized below.

**Table 1:** Steps 1–7 and related methods and sources of information

Step	Attributes	Methods and sources of information
Step 1. Food system boundaries	- Problem-specific boundaries - Geographical boundaries (local, regional, national, global) - Product/commodity (fruits, tomato, livestock)	- Multi-stakeholder workshops - Secondary sources (e.g., reports, and statistics) - Expert interviews
Step 2. Agricultural production system types	a) Structural variables b) Functional variables	- Expert-based methods - Multivariate analysis - Surveys - Secondary sources (census data and statistical reports)
Step 3. Value chain types	a) Network structure - Horizontal and vertical relationships b) Value chain governance - Bilateral contracts - Network governance - Informal mechanisms	- Value chain mapping - Qualitative and quantitative indicators - Interviews with value chain actors - Multi-stakeholder workshops - Secondary sources (e.g., reports, scientific literature)
Step 4. Support structures innovation and functioning agricultural production systems and value chains	a) Economic and innovation policies and instruments b) Private and public Research and Development (R&D) programs c) Private and public extension approaches	- Multi-stakeholder workshops - Expert interviews - Surveys - Secondary sources (e.g., policy documents, reports, policies, scientific literature)
Step 5. Food system typology	Food system = agricultural production system type + value chain(s) type(s) + encompassing support structures	Synthetizing and combining information from steps 2 to 4
Step 6. Food system outcomes	Contribution of a food system to a) Food security and nutrition b) Environmental security c) Social welfare	- Literature review - Multi-stakeholder workshop - Expert-based assessments - Empirical data
Step 7. Classification of food systems	Dominant food systems, niche food systems, hybrid food systems	- Market share data

### Step 1: identifying the food system and defining the system boundaries

In this first step the boundaries and the level of detail of the study are defined. The boundaries may delimit the food system for a particular food commodity (e.g., tomato, all fruits, and vegetables) and/or for a specific geographic area (e.g., region or country). The food system regime and the dominant food systems, niche food systems, and hybrid forms are defined within these boundaries. Anything outside the boundaries is, by definition, considered part of the exogenous socio-technical landscape within the multi-level perspective (Avelino 2017). This step determines the scope of the study and is therefore closely linked to the nature of the problem that is to be analyzed (Neshiem et al. 2015). When identifying the boundaries and the level of detail of the study, it is also fundamental to take questions of political economy into account and, in doing so, identify dynamics such as gender, class, power, and access to resources.

Food systems may be connected to socio-technical regimes outside of their ostensible boundary (e.g., health, tourism, and energy sector). This diffuse or context-dependent nature of the boundaries makes defining and delineating the food system regime and the food systems not straightforward. Therefore, the initial choice of boundaries should be revisited and adjusted as an inherent part of the implementation of the framework.

### Step 2: identifying agricultural production system types

This step consists of setting up agricultural production system typologies. Although agricultural production systems are dynamic, production system typologies can give snapshots of farm diversity at certain moments in time (Alvarez et al. 2014). Production system typologies can be grouped into two main classes (Alvarez et al. 2014). Structural typologies, which are based mainly on variables that describe resources and asset levels, include variables such as area, number of cattle, hired and family labor, and available irrigation water. Functional typologies are based on variables that describe livelihood strategies and household dynamics such as production orientation and sources of income (Tittonell 2014b in Alvarez et al. 2014). The combination of structural and functional variables would often be needed in the construction of agricultural production system typologies.

To construct structural and functional agricultural production system typologies, various methods may be used, ranging from expert-based methods in which agricultural production systems are aggregated into clusters defined by local experts, key informants, and producers (Alary et al. 2002; Kuivanen et al. 2016) to multivariate analysis supported by statistical techniques (Alvarez et al. 2014; Pacini et al. 2014). Multivariate statistics methods are commonly preferred over expert-based approaches due to the structured approach for analysis and greater reproducibility. However, expert-based approaches can enhance the relevance of typologies to stakeholders. Therefore, using both approaches in a complementary way is recommended (Alvarez et al. 2014).

### Step 3: identifying the types of value chains associated to agricultural production system types

This step consists of identifying and describing the value chains that link each of the agricultural production system types identified in step 2 to markets and consumers. Value chains can be characterized based on their network structure and their governance form (Trienekens 2011). Description of the network structure involves the identification of the value chain actors, including consumers, which are linked to each of the agricultural production system types and how they relate vertically and horizontally. The value chain structure and its link to each agricultural production system type (or its commodity) can be visually represented using value chain mapping (Herr and Muzira 2009). Governance forms in vertical and horizontal relationships can be elucidated based on transaction costs and value chain and network theory. Following Trienekens et al. (2018), three elements in value chain governance can be distinguished. First, bilateral contracts throughout the chain and their coordination mechanisms on price, volume, time of delivery, and quality. Quality is described by intrinsic product attributes such as color, safety, tenderness and taste, and extrinsic characteristics which cannot be tangibly measured but that are embedded in conventions of trust, tradition, nature, and place (Goodman 2003). Second, the network governance in which lead parties, shared governance, and value chain facilitation are key elements of the governance structure. Third, informal coordination mechanisms such as trust, reputation, power, and commitment. These three value chain governance elements can be operationalized by means of qualitative and quantitative indicators.

### Step 4: identifying the multiple setups of support structures

This step aims at identifying the structures for support of innovation and everyday functioning of agricultural production systems and value chains. Main structures of support include research and development (R&D), extension services, and innovation policies. *R&D* underpins policies and innovations by providing knowledge, data, and novel practices (Wesley and Faminow 2014). R&D activities can be undertaken by public research institutes and universities, the private sector (e.g., agribusiness companies looking for adequate business models for sustainable food production), and by public-private partnerships. *Extension services* refer to the set of public and private organizations and institutions that support agricultural producers and value chain actors in solving problems and obtaining information, skills, and technologies to improve the sustainability of their operations. Multiple models for extension may exist, including top-down and paternalistic; supply-driven; demand-driven, participatory, and pluralistic; technology-driven; and gender-sensitive (Wesley and Faminow 2014). In our definition of extension services, grassroot knowledge-sharing systems are also an important element. These systems refer to alternative and horizontal forms of producing, organizing, and exchanging of information. *Economic and innovation policies* refer to the set of policies and instruments (e.g., subsidies, fiscal incentives, and policies for training and skills) that contribute to innovation in agricultural production systems and value chains.

Innovation policies can be divided in (i) mission-oriented policies, aimed at providing practical solutions to specific sustainability challenges; (ii) intention-oriented policies, which concentrate on the R&D; and (iii) system-oriented policies, which focus on system-level features, such as the degree of interaction between different parts of the food system (agricultural production systems and value chain actors). Instruments for innovation policy include, among others, fiscal incentives for R&D, direct support to R&D and innovation, policies for training and skills, polices to support collaboration, innovation network policies, standards, regulations, and technology foresight (Edler and Fagerberg 2017).

To identify the support structures different information sources can be used including literature review, multi-stakeholder workshops, interviews and surveys with food system actors, and secondary sources (policy documents, project reports, laws, curricula for agricultural education and training).

### Step 5: identifying the diversity of food systems

In this step, the findings from steps 2, 3, and 4 are synthesized to characterize the diversity of co-existing food systems. Each of the food systems is constituted by the interrelation between an agricultural production system type, its associated value chain(s), and the encompassing support structures.

### Step 6: assessing food system outcomes

This step consists of measuring performance of the multiple food systems identified in step 5 in terms of food security and nutrition, environmental security, and social welfare. Measuring performance of a food system in terms of the three food system goals requires operationalization through indicators. Indicators may be drawn from existing studies and reports, which can allow for comparability with previous research. Examples include the set of indicators on healthy diets and sustainable food systems developed by the EAT initiative, the Sustainable Development Solutions Network (SDSN) and the CGIAR Consortium (EAT initiative 2015); the Household Food Insecurity Access Scale (HFIAS) indicators (Coates et al. 2007); and indicators in the Global Nutrition Report 2015 of the International Food Policy Research Institute (IFPRI 2015), the FAO food security indicators (FAO 2017) and the Sustainability Assessment of Food and Agriculture systems (SAFA) tool (FAO 2014b). The suite of indicators should be selected based on the socio-economic and environmental context to which the framework will be applied.

### Step 7: classifying food systems through the lens of the MLP

The first six steps of the framework allowed the characterization of the existing diversity of food systems. With the diversity of food system configurations characterized, the attention in this step turns to classifying food systems in terms of being dominant, niche food systems or hybrid forms. This information can make visible the undervalued and marginalized niche and hybrid food systems and can assist and support policy makers and stakeholders in the design of strategies to stimulate and induce developments towards a desired sustainability-enhancing pathway.

To classify the food systems as dominant food systems, niche food systems, and hybrid systems, the indicator market share in terms of sales and production volume is proposed as a proxy of the level of alignment of each food system to the food system regime. The food systems identified in step 5 with the largest market shares and/or production volumes are classified as being part of the food system regime, thus as dominant food systems. Using these dominant food systems as the benchmark, the remaining food systems are distinguished based on the level of deviation in organization and practices. The larger the deviation is, the more radical the food systems are.

An alternative and complementary tool to classify food systems is the typology of organizational relations adapted by Duncan and Pascucci (2017) from Grandori and Furnari (2008). Following this typology, food systems involving relations that are predominantly bureaucratic and/or market-based, i.e., food systems with isomorphic organizational relations, tend to be aligned to the food system regime. On the other hand, food systems that are built around community and democratic relations, i.e., food systems with polymorphic organizational relations, tend to provide the space and conditions for experimenting with radical practices that are less likely to conform with those facilitated by the food system regime.

Having presented the methodological approach for implementation of the framework, in the next sections we provide an illustrative example of how our framework can be operationalized in real life situations (steps 1–7), and we reflect on its usefulness, shortcomings, and further development and use.

## Illustration to vegetable food systems in Chile: lessons from the application of the framework

To illustrate how the framework may be operationalized, we present an illustrative example aimed at characterizing the diversity of vegetable food systems in Chile, and analyzing their potential to harness production of vegetables with low-or-no pesticide use. Chile and its prevailing agro-export development model that was started 35 years ago (Ríos-Núñez 2013) is an example of how the global corporate-(environmental) food regime manifests in a country. Government and industry strategies in food production and commercialization are largely relying on technical innovation, efficiency, and productivity, using the common denominator of sustainable food production. Efficiency in the use of natural resources and minimization of negative externalities is put forward as the approach to limit environmental damage (Martínez-Torres et al. 2017). Nowadays, the vegetable sector of the Chilean food system regime experiences several socio-technical landscape pressures related to the pesticide use: (i) the increasing social concern and awareness over the impact of pesticides on the environment and on human health (Martínez-Torres et al. 2017); (ii) the increasing international prices of chemical pesticides, which create uncertainty over the long-term feasibility of the current approach of food production; (iii) increasingly informed consumers demanding healthier food (Martínez-Torres et al. 2017); and (iv) government commitments to meet national and international targets on pesticide residues in food. As result of these pressures, multiple incremental and radical innovations are being developed and adopted in food systems. In this example, we aimed at characterizing the diversity of co-existing vegetable food systems in Chile in order to identify those systems with potential to harness production and commercialization of vegetables with low-or-no pesticide use. As vegetables in Chile are mainly produced for and marketed on the national market (ODEPA 2015), the national level was chosen as the study’s geographical boundary (step 1 of the framework).

Drawing on this illustrative example, we reflect on usefulness, shortcomings, and further development and use of the framework.

### Data collection and analysis

Data to characterize and classify vegetable food system types were gathered from June 2017 to August 2017 through 33 semi-structured in-depth face-to-face interviews, complemented with data gathered from published reports, studies and documents, and field observation. The steps of the framework (see Sect. 3) were used as guideline for the semi-structured interviews. Interviewees were purposely selected as persons with key knowledge on of the vegetable sector in Chile, either regionally and/or nationally. Interviews were recorded, transcribed, coded, and analyzed with reference to the construction of vegetable production system types, value chain types, and setups of support structures. Interviewees and their placement on the food system component and on the MLP are presented in Table 2.

**Table 2:** Interviews and placement of actors based on the food system component and on the lens of the multi-level perspective (MLP) framework

Food system component	Institution/activity	Placement on the MLP
Agricultural production system	Regional horticultural program Large conventional producer Organic large scale producer Medium organic producer Agroecological producer 1 Agroecological producer 2 Community-supported agriculture Agroecological community Association ecological producers	Regime level Regime level Regime/niche level Niche level Niche level Niche level Niche level Niche level Niche level
Value chain	Wholesale market Lo Valledor Street markets (ASOF) AFIPA Eco-fair 1 Eco-fair 2 Eco-shop Intermediary/distributor 1 Intermediary/distributor 2	Regime level Regime level Regime level Niche level Niche level Niche level Niche level/regime level Niche level
Support structures	Extension services INIA—organic transfer group INDAP program local development Private advisor Research Researcher University of Chile Researcher University of Valparaiso Ministry of Agriculture INDAP sustainability program INDAP commercialization program SAG organic agriculture/certification SAG organic agriculture/inputs ACHIPIA ODEPA FIA Ministry of Economy and Development CORFO Innova Chile Sustainability and Climate Change Agency ProChile	Regime level Regime level Regime/niche level Regime level Regime level Regime level Regime level Regime level Regime level Regime level Regime level Regime level Regime level Regime level Regime level Regime level

*ASOF* National Trade Union Confederation of Street Markets, *AFIPA* association of manufacturers and importers of phytosanitary products, *PRODESAL* Program of Local development, *INDAP* Institute for Agricultural Development, *ODEPA* Office of Agricultural Studies and Policies, *FIA* Foundation for Agricultural Innovation, *ACHIPIA* Chilean Agency for Food Safety, *SAG* Agricultural and Livestock Service, *INIA* Agricultural Research Institute, *CORFO* Corporation for the Promotion of Production, *ProChile* Chile’s Export Promotion Agency

Agricultural production system types were constructed following an expert-based typology (step 2 of the framework). As agricultural production systems that are based on ecological intensification (EI), such as organic farming, agroecology, and diversified farming systems, have been proposed as promising radical sustainability innovations to reduce or eliminate pesticide use in food production by making intensive and smart use of the natural functionalities of the ecosystems (Tittonell et al. 2016), the characterization of vegetable food systems was focused on a gradient of EI, ranging from conventional production systems based on (regulated) pesticide use to production systems where intentions, regulations, and practices are to avoid pesticides. Table 3 presents the qualitative variables used to identify and describe the vegetable production system types.

**Table 3:** Qualitative variables for characterizing vegetable production systems along a gradient of ecological intensification (EI)

Characteristic	Variable	Unit
Size	Total area of farm	Hectares
Labor	Family labor	Proportion
	Hired labor	Proportion
EI practices/agronomic management	Use of fertilizers and pesticides/dependence on external inputs, use compost, use of bio-control agents, crop rotations, and diversification	Yes/no

Value chain types that link the vegetable production system types to consumers were described in terms of their network structure (vertical and horizontal relationships) and their governance form (step 3 of the framework). Table 4 presents the qualitative variables used to characterize the value chain types.

**Table 4:** Qualitative variables for characterizing value chains that link vegetable production system types to markets and consumers

Characteristic	Variable	Description
Network structure	Vertical relationships	Collaboration between actors in different activities of the value chain
	Horizontal relationships	Collaboration between actors in the same activity of the value chain
Value chain governance	Safety	Actor setting and controlling safety requirements/scope of the safety requirements
Network governance	Shared governance ^a^	Frequency of meetings between members, participation in decision-making
Informal mechanisms	Trust	Low, medium, high

^a^There are other governance forms of horizontal and vertical relationships in value chains. For example, lead organization governance and network-administrative governance. For a detailed description, see Provan and Kenis (2008)

The support structures, including research and development (R&D) programs, extension services, and innovation policy, were identified for each combination vegetable production system type and value chain type (step 4 of the framework). Emphasis was put on those structures that have an influence on the use and control of pesticides, e.g., training, organic labels, and food safety standards.

Data gathered for steps 2 to 4 was synthetized to characterize vegetable food system types (step 5 of the framework).

Once diversity of vegetable food systems in Chile was characterized, a preliminary assessment of the outcomes of the various food system types was made through a questionnaire among seven experts with knowledge on the vegetable sector in Chile (step 6 of the framework). Stakeholders were approached through email and by personal contact. Four of the stakeholders were different than the interviewees. We asked the stakeholders to evaluate each vegetable food system type by providing their degree of agreement on a Likert scale with a list of 18 statements. The statements considered the three final outcomes of the food system: food security and nutrition, environmental security, and social welfare, which includes society and economy. The statements were built based on the scientific literature on the principles and values that underpin a sustainable food system (Bajagai 2014; EAT initiative 2015; FAO 2014a, b; Gustafson et al. 2016; IPES-Food 2015; IFPRI 2015; Peano et al. 2015). Example statements for each food system outcome are provided in Table 5.

**Table 5:** Example of statements for the evaluation of food system outcomes: food and nutrition security, environmental security, and social welfare

Food system outcome	Statement
Food and nutrition security	The vegetable food system provides vegetables whose prices are accessible to all consumers in Chile, regardless of their socio-economic level
Environmental security	The vegetable food system reduces or eliminates the release of pesticides on the environment
Social welfare—society	The vegetable food system is economically profitable (overall)
Social welfare—economy	The vegetable food system encourages consumers to know where, how, and who produces their vegetables

Finally, the vegetable food systems were classified as dominant food systems, niche food systems, and hybrid forms using the indicator market share in terms of volume (step 7 of the framework).

Results of this illustrative example are part of a diagnostic study of the vegetable sector in Chile undertaken within the 4-year NWO-funded project HortEco. A more extensive report on this example is being prepared for publication (Gaitán-Cremaschi et al. 2018).

### Characterization of vegetable food systems in Chile and food system outcomes

Synthesis of the data gathered for step 2 to step 4 resulted in a typology of five vegetable food system types. The characteristics of each of the types are described in Table 6. Here, we present a summary of these characteristics.

**Table 6:** Main characteristics of vegetable food system types in Chile: agricultural production systems + associated value chains + setup of support structures, and food system outcomes

Characteristics	Type I	Type II	Type III	Type IV	Type V
Vegetable production systems					
Area	< 12 ha HRB	< 12 ha HRB	< 20 ha	> 20 ha	> 12 ha HRB–100 ha
Family labor	+++	+++	++	–	+/−
Hired labor	+/−	+/−	+	+++	+++
Level of EI	+/− to +	+ to +++	++ to +++	+/− to +	− to +/−
Value chains					
Vertical relationships	+/−	+/− to ++	+ to ++	+/− to +	+/− to +
Horizontal relationships	+/−	+/− to ++	+ to ++	–	+/−
Strictness contract (safety)	–	+/−	+++	+++	+
Shared governance	–	− to +	++	+/−	− to +/−
Trust	–	− to ++	++	+/− to +	− to +
Structures for support of innovation and functioning					
R&D	Formal education centers and public research centers such as INIA	NGOs, mainly alternative research centers, grassroot networks and social movements (learning by-doing)	NGOs, mainly alternative research centers, grassroot networks and social movements (learning by-doing)	Private research centers and learning by doing. Formal education centers and public research centers (marginal)	Formal education centers and public research centers (e.g., INIA)
Extension services	Extension financed primarily by INDAP and delivered by privates. Technical advice input supplying companies Pesticides: extension focused on management and disposal of pesticides, GAP and CP agreements (marginal)	Grassroot knowledge-sharing systems, NGOs, alternative research centers. Extension primarily financed by INDAP and delivered by privates. Public extension in agroecology (marginal)	Grassroot knowledge-sharing systems, NGOs, alternative research centers. Public extension in organic agriculture (marginal)	Private extension with a demand-driven approach and input sellers providing technical advice	Private extension with a demand-driven approach + technical advice input supplying companies Pesticides: extension focuses on pesticide efficiency, GAP and CP agreements
Innovation policy	Public policies, programs and funding through INDAP	Innovation comes from grassroot networks and social movements. Limited public policies, programs and innovation in agroecology and commercialization	Law on organic production/Innovation comes from grassroot networks and social movements. Limited public policies on organic production and commercialization	Law on organic production. Public innovation agencies and development programs (FIA, CORFO, etc.). Not specific for organic vegetable production and commercialization	Public policies Ministry of Agriculture (FIA) and the Ministry of Economy and Development (CORFO) (not specific for the vegetable sector)
Food system outcomes^a^					
Food security and nutrition	2.1	2.6	2.8	3.0	3.8
Environmental security	1.0	4.2	4.5	3.9	1.7
Social welfare (society/economy)	1.2/2.0	3.2/3.1	3.8/3.5	3.0/3.7	2.7/3.8

Type I: resource constrained conventional vegetable food system, type II: agroecological vegetable food system, type III: Locally embedded organic vegetable food system, type IV: business-oriented organic vegetable food system, type V: medium-large business-oriented conventional vegetable food system

+++ means very strong/very high, ++ means strong/high, + means moderate/medium, +/− means limited/low, − means lack of/very low

HRB hectare of basic irrigation, GAP good agricultural practices, CP clean production

^a^Food system outcomes based on a six-point Likert scale, where 0 represents a strongly negative performance and 5 a strongly positive performance (average scores)

*Type 1—resource-constrained conventional vegetable food systems* characterized by small-sized production units with conventional management using fertilizers and pesticides, and some traditional farming practices, resulting in very low to medium levels of EI. These systems connect to consumers mostly through the traditional marketing channel i.e., farmer, intermediaries in wholesale markets, small retailers and consumers. Due to abundant and largely uncontrolled pesticide use food safety is low. *Type II—agroecological food systems* characterized by small-sized production units managed agroecologically with occasional use of synthetic pesticides and fertilizers, resulting in medium to very high levels of EI. Products reach consumers through multiple short marketing channels such as local fairs, specialized shops, farmer marketing cooperatives, home delivery, and farm gate sales, or through the traditional marketing channel. Food safety is in most cases not controlled and relies on proximity relations and trust. *Type III—locally embedded organic vegetable food systems* characterized by small- to medium-sized production units managed agroecologically, resulting in high to very high levels of EI. Certified organic production takes place through the Participatory Guarantee System (PGS). Farmers market the products individually through specialized shops, home delivery, and farm gate sales, or in association through PGS marketing cooperatives and eco-fairs. *Type IV—business-oriented organic vegetable food systems* characterized by medium- to large-sized production units managed with commercial organic pesticides and organic fertilizers, resulting in low to medium levels of EI. Organic certification is achieved through external certification bodies authorized by the Chilean government. Products are mostly distributed through supermarket chains and to a lesser extent through specialized shops, eco-fairs, and organic markets. *Type V—medium-large business-oriented conventional food systems* characterized by medium- to large-sized production units with conventional management (synthetic pesticides, mono-cropping, and intensive tillage), resulting in very low to low levels of EI. At medium-sized farms the products are mostly commercialized trough the traditional marketing channel. At larger farms products are mostly commercialized through the agroindustry and the retail. Pesticide use is partially monitored by supermarket chains, the agroindustry, and governmental bodies.

Stakeholder assessment of food system outcomes showed good performance of vegetable food systems type II and type III in social terms (scoring 3.2 and 3.8 out of 5 on the Likert scale) and environmental terms (scoring 4.2 and 4.5 out of 5 on the Likert scale), in line with ambitions of the actors who envision more ecological and inclusive systems. Lower performance was found in terms of food security and nutrition (scoring 2.6 and 2.8 out of 5). The stakeholders noted that the systems type II and type III provide an insufficient volume of vegetables to meet the national demand and that the vegetables cannot be purchased everywhere in Chile. Vegetable food system type V performed well in terms of food security and nutrition (scoring 3.8 out of 5), as well as in economic terms (scoring 3.8 out of 5). Built with a predominantly economic focus, this food system received low scores on environmental and societal outcomes (scoring 1.7 and 2.7 out of 5). Vegetable food system type IV received medium scores for societal outcomes and food security and nutrition outcomes (scoring 3.0 out of 5), and received high scores on economic benefits (scoring 3.7 out of 5). Vegetable food system type I performed poorly in the three food system outcomes.

The characterization process allowed to identify a preliminary typology of vegetable food systems in Chile and the characteristics that may harness production and commercialization of low-or-no-pesticide vegetables. Each of the five system types could be further disaggregated. For example, the medium-large business-oriented conventional vegetable food system could be further disaggregated into subtypes based on strategies of pesticide use (e.g., good agricultural practice), and all types could be disaggregated based on biophysical setting of the farms or nature of contracts.

A shortcoming of our framework, which is inherent to general problems of qualitative as well as quantitative typologies, is to aggregate observations into homogenous juxtaposed compartments. For the case at stake, this may hide the fuzzy, partly overlapping spaces among the five vegetable food systems.

### Classification of vegetable food systems in Chile

Analysis of the indicator market share in terms of volume revealed that the vegetable food systems type I and type V in Chile can be classified as dominant food systems. These types comprise almost 100% of total volume of the vegetable products that are marketed nationally. It is estimated that the retail industry has a share of around 17% of the commercialization of vegetables and the traditional marketing channel (especially through the wholesale market of Lo Valledor) a share of almost 83% (Boitano-Contreras 2011; Schwartz et al. 2013). These two types are characterized by isomorphic relations, grounded in quality standards and requirements set by supermarket chains and the agroindustry, and formalized participation rules such as entry fees in wholesale markets, and minimum volumes and qualities. On the other hand, the vegetable food system types II, III, and IV together do not reach 1% of total volume. Of these three types, type II and type III can be classified as niche vegetable food systems. Both systems commonly organize themselves around networks of producers and consumers (e.g., agroecological communities, associations of ecological producers, PSG cooperatives, and associations of responsible consumers), based on relations of trust, collaboration, transparency, and equity. These systems respond to pressures of the socio-technical landscape by promoting the reconnection between production and consumption, giving greater attention to local and ecological vegetable production against the industrial principles of the Chilean food system regime. Food system type IV can be classified as a hybrid system closely aligned to the food system regime. This system maintains practices and organizational forms supported by the Chilean food system regime while it incorporates practices of organic agriculture and agroecology such as crop rotations and in a few cases diversification, which are stimulated by a growing concern for the environmental integrity and the consumption of “healthy” products. This system promotes the so called “corporate greening” focusing on the claim of healthiness of vegetables (Ríos-Núñez and Núñez-Yáñez 2016), and seeks to tailor organic production to the conventions of industrial agriculture and the industrial market.

For the case at stake, the indicator market share of total volume allowed a locally recognizable classification of vegetable food systems as dominant vegetable food systems, niche vegetable food systems, and hybrid forms. Nevertheless, it is worth noting that depending on the specific socio-economic context to which the framework is applied, the information provided by these indicators may hide the level of development of niche food systems. For example, in developing countries, commercialization of niche food products through multiple short circuits is crucial for local development and food security. However, the importance of the type of markets that are behind these short circuits commonly are absent from the official statistics. Therefore, additional indicators to capture the level of alignment of food systems to the regime may need to be developed.

Following the national level example described here, research efforts may be focused on supporting food systems types II, III, and IV. A renewed application of the framework may elucidate diversity within each of these vegetable food system types. Moreover, this preliminary illustration could be further developed particularly when it comes to the evaluation with actors. The example of Chile has been elaborated on the basis of interviews, secondary sources of information, and field observations but has not been fed back to the actors. Evaluation workshops would contribute to triangulation of results and enable their use by stakeholders active in the transition to a more sustainable vegetable sector.

## Conclusions

Numerous studies address food system transitions. However, an operational approach to map the diversity of food systems to reveal patterns of more and less sustainable characteristics is still lacking. We proposed a framework developed to serve characterization and analysis of the diversity of food systems in a defined geographical area (e.g., region or country), and to structure thinking about possible changes to the status quo from within or outside mainstream food systems. The framework enables bringing together information from disparate knowledge domains in science and practice (such as concepts in agronomy, value chain management, innovation systems, food system governance, and environmental sciences) to support reflection, decision making, and informed discussion on bringing about changes towards more sustainable ways of food production, marketing, and consumption. Moreover, the framework allows to structure thinking about how to assess food system performance as a basis for informed decision making. By distinguishing major structural elements of food systems, it may help to bring out developments and alternative food systems to dominant ones that, while potentially important to the achievement of food system goals, remain invisible in statistical data. For example, the results of the illustration were particularly useful to identify vegetable food systems that are not well distinguishable in market and volume statistics, but may have potential for instigating change. Implementation of the framework can be deepened by social network analysis to identify the role of change agents and actors in the development and adoption of sustainable innovations in food systems.

Although the characterization process simplifies the complexity of food systems, results of the framework can be used to focus attention on functioning of the food system components and reveal main barriers and promising elements. Agent-based modeling (Matthews et al. 2007; Utomo et al. 2018; Van Dam et al. 2012) in combination with design-oriented approaches (Ballantyne-Brodie and Telalbasic 2017) may be used to integrate information and provide leverages for sustainability transitions. Results of the framework can be used in the exploration of potential transition pathways of food systems. For this purpose, results can inform the development of socio-technical scenarios, which have been widely used to explore consequences of alternative futures on transitions, especially in the energy and transport sector (e.g., Elzen et al. 2002; Foxon et al. 2010; Foxon 2013; Shackley and Green 2007; Verbong and Geels 2010). Rather than aiming for prediction of developments from within or outside the food system regime, the aim of the scenarios would be at exploring the conditions and niche-regime interactions that are necessary for the realization of different transition pathways (see, for example, Geels et al. 2016 for a typology of transition pathways). Such information can be used strategically to assist and support actors and decision makers in the realization of a given desired pathway (see also Hebinck et al. 2018).

Given the multi-functional nature of food systems, a further development of the framework could be to include the interactions between the food system regime and socio-technical regimes pertaining to other sectors, e.g., interactions between the food system regime and the energy system, health system, tourism system socio-technical regimes (Geels 2011; Hassink et al. 2013; Pigford et al. 2018; Sutherland et al. 2015a, b). For example, the growth and upscaling of radical innovations such as those in the vegetable food systems type II and type III of our illustration would require of coalitions and alliances with actors in two (or more) of these socio-technical regimes.

Finally, a further extension of our framework could include a common method for the comparison of the food system regime and the diversity of food systems across geographical scales (following Fuenfschilling and Binz 2018). For example, a method that affords to look at different countries and answer questions such as how does the food regime manifest in national food system regimes; what are the existing dominant systems, niche systems, and hybrid forms in the different countries; and what are the shared features of these systems? Comparative analysis of food system dynamics in different countries may contribute to giving more nuanced views on the diversity of sustainability trajectories, following a plea by Friedmann (2016) that the debate on food regimes needs to be widened.
